# Mincle-mediated anti-inflammatory IL-10 response counter-regulates IL-12 *in vitro*

**DOI:** 10.1177/1753425916636671

**Published:** 2016-03-02

**Authors:** Emmanuel C Patin, Sam Willcocks, Selinda Orr, Theresa H Ward, Roland Lang, Ulrich E Schaible

**Affiliations:** 1 Department of Immunology and Infection, Faculty of Infectious and Tropical Disease, London School of Hygiene and Tropical Medicine, London, UK; 2 Priority Area Infections, Research Center Borstel, Borstel, Germany; 3 Institute of Infection and Immunity, School of Medicine, Cardiff University, Cardiff, UK; 4 Institute of Clinical Microbiology, Immunology and Hygiene, University Hospital Erlangen, Friedrich-Alexander-Universität Erlangen-Nürnberg, Erlangen, Germany; 5 German Center for Infection Research, TTU-TB, Borstel, Germany

**Keywords:** Mycobacteria, Mincle, IL-10, macrophage, receptor

## Abstract

The role of macrophage-inducible C-type lectin (Mincle) in anti-inflammatory responses has not yet been fully characterized. Herein, we show that engagement of Mincle by trehalose-dimycolate or mycobacteria promotes IL-10 production in macrophages, which causes down-regulation of IL-12p40 secretion. Thus, Mincle mediates both pro- as well as anti-inflammatory responses.

## Introduction

Better understanding of innate immunity to mycobacteria is critical to develop novel vaccination concepts to control worldwide tuberculosis (TB) incidence. Innate immune responses are primarly orchestrated by macrophages and dendritic cells, which produce cytokines upon microbe-associated molecular pattern (MAMP) recognition by PRRs, including C-type lectin receptors and TLRs.^1^ The pro-inflammatory cytokines TNF-α and IL-12 are important for protective immunity against mycobacterial infection in mice, particularly by induction, expansion and regulation of CD4+ Th1 cells.^2^ In contrast, IL-10 counter-regulates inflammation and over-expression of IL-10 increases susceptibility of mice to mycobacterial infections.^2^

The macrophage-inducible C-type lectin (Mincle; CLEC4E) receptor activates the NF-κB signalling pathway following Fc gamma (Fcγ) chain-spleen tyrosine kinase (Syk) recruitment.^3^ Mincle acts as receptor for the mycobacterial cell envelope glycolipid trehalose dimycolate (TDM).^4,5^ Mincle mediates pro-inflammatory cytokine production, as well as effector killing mechanisms, including NO synthesis upon interaction with either TDM or mycobacteria.^4,5^ Pro-inflammatory cytokine production upon injection of *Mycobacterium bovis* BCG or TDM into mice was impaired in mincle-deficient animals.^4,6,7^ TLR2 has also been shown to sense MAMP, such as triacylated and diacylated lipoproteins, from mycobacteria.^8^

The role of Mincle in anti-inflammatory responses to mycobacteria has not yet been studied. Here, we show that TDM modulates TLR2-mediated IL-10 and IL-12p40 responses in macrophages through Mincle, which is, in turn, up-regulated by BCG. These findings have important implications for function and design of anti-TB vaccines and adjuvants in subunit vaccine formulations.

## Materials and methods

### Bone marrow-derived macrophages

Bone marrow-derived macrophages (BMDM) were prepared from C57BL/6 WT, Mincle^–/–^, Fcγ^–/–^, IL-10R^–/–^ and TLR2^–/–^ mice as previously described.^9^ TLR2^–/–^ mice were a kind gift from Marina Freudenberg (Max-Planck Institute of Immunobiology and Epigenetics, Freiburg, Germany).

### Preparation of TDM-coated beads and mycobacteria

TDM-coated and control BSA beads were prepared and BCG Pasteur was grown in 7H9 as previously described.^9,10^ TDM from BCG was purchased from Bioclot (Germany).

### Quantification of cytokines and NO

BMDMs were incubated with beads, BCG, LPS (Sigma-Aldrich, Dorset, UK), trehalose-dibehenate (TDB), Pam_3_CSK_4_ (Invivogen, UK) or recombinant mouse IL-10 (R&D Systems, Abingdon, UK) as indicated. Two hundred nM Syk selective inhibitor (imidazopyrimidine; Santa Cruz Biotechnology, Santa Cruz, CA, USA) was given prior to infection. Supernatants were collected for cytokine and NO analysis at indicated time points. NO, TNF-α, IL-10 and IL-12p40 were measured by Griess reaction (Sigma-Aldrich) or ELISA kits (BD Biosciences or R&D Systems), respectively.

### Western blot

Wild type BMDMs were infected with BCG Pasteur in D10 medium at a multiplicity of infection (MOI) of 10 or left untreated for 24 h. Western blot from cell lysates was performed using primary rat anti-Mincle (4A9; MBL International, Woburn, MA, USA), as well as secondary HRP-conjugated goat anti-rat (Jackson ImmunoResearch, West Grove, PA, USA) Abs. Signals were detected using the ECL Advance Chemiluminescence kit and ECL Hyperfilm (GE Healthcare, Little Chalfont, UK) according to the manufacturer's instructions.

### Statistics

Data represent means ± SEM of averages from three or four independent experiments. One or two-way ANOVA followed by Bonferroni’s post-test was used for statistical analysis when multiple groups were analysed and Student’s *t*-test when two groups were analysed. Data were considered significant when *P* < 0.05.

## Results

### Mincle modulates TLR2-mediated pro- and anti-inflammatory responses in macrophages

**Figure 1.**
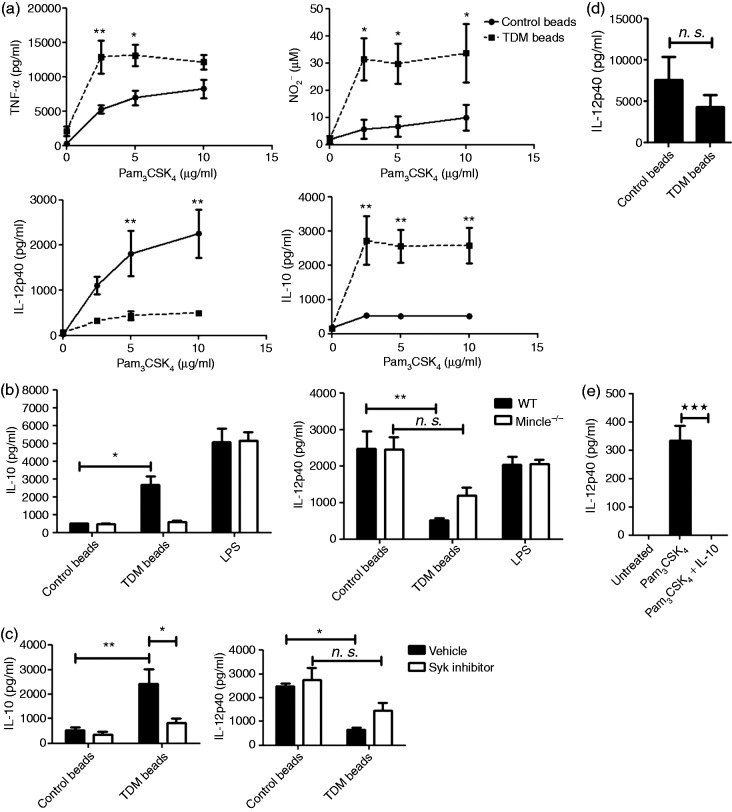
Induction of IL-10 production in macrophages requires Mincle and TLR2 signalling. (a) WT BMDMs (10^5^) were co-stimulated with 106 control or TDM-coated beads (50 µg lipid/10^7^ beads) and Pam3CSK4 at the concentrations indicated for 48 h. WT or Mincle^–^/^–^ (b), WT (c) or IL10R^–^/^–^ (d). BMDMs (10^5^) were co-stimulated with 10^6^ control or TDM-coated beads (50 µg lipid/10^7^ beads) and 10 µg/ml Pam3CSK4 or stimulated with LPS (1 µg/ml) for 48 h. (e) WT BMDMs were stimulated with 10 µg/ml Pam3CSK4 with or without 1 ng/ml rmIL-10 for 48 h. Where indicated (c), BMDMs were incubated with 200 nM Syk inhibitor for 30 min prior to co-stimulation. TNF-α, IL-12p40, and IL-10 or NO (${\text{NO}}_{2}^{-}$) concentrations were analysed in supernatants by ELISA or Griess assay, respectively. Data are expressed as means ± SEM from three [a, b, c (IL-12p40), e] or four [c (IL-10) and d] independent experiments done in triplicate [two- (a) or one-way (b, c and e) ANOVA followed by Bonferroni’s post-test and (d) Student’s *t*-test, *P < 0.05; **P < 0.01; ***P < 0.001; n. s.: not significant].

To mimick interaction between Mincle and TDM exposed at the mycobacterial cell envelope, TDM was coated to beads.^9^ We observed a significant increase in TNF-α, NO and IL-10 production following co-stimulation of BMDMs with TDM beads and Pam_3_CSK_4_. In contrast, TDM beads dampened Pam_3_CSK_4_-mediated IL-12p40 secretion (Figure 1A).

In the absence of Mincle, as well as upon inhibition of Syk, IL-10 responses to TDM beads/Pam3CSK4 were abolished (Figure 1B, C). Lack of Fcγ also abolished IL-10 secretion in response to TDB/Pam_3_CSK_4_ (Figure S1). More importantly, inhibition of Pam_3_CSK_4_-induced IL-12p40 production by TDM was less pronounced in macrophages lacking Mincle or treated with Syk inhibitor (Figure 1B, C). To analyse whether TDM/Pam_3_CSK_4_-induced IL-10 down-modulates IL-12p40 responses, IL-10R^–/–^ BMDMs were incubated with a combination of TDM beads and Pam_3_CSK_4_. TDM was unable to interfere with Pam_3_CSK_4_-mediated IL-12p40 production in the absence of IL-10 signalling in a statistically significant manner (Figure 1D). Furthermore, the addition of exogenous IL-10 to macrophages concomitantly with Pam_3_CSK_4_ completely inhibited IL-12p40 production (Figure 1E). Taken together, engagement of Mincle modulates TLR2-mediated IL-12p40 responses primarily through IL-10.

### BCG up-regulates Mincle expression in macrophages independent of TLR2

**Figure 2.**
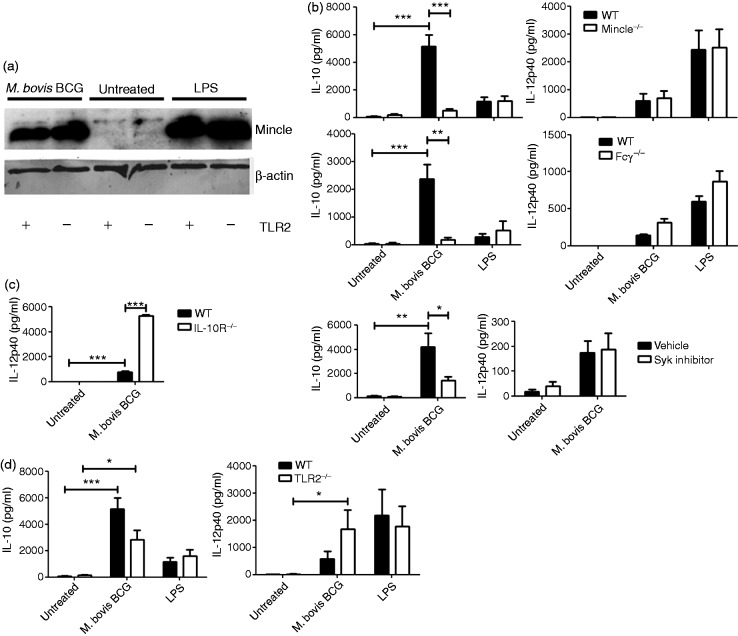
Live *M. bovis* BCG induces IL-10 production through Mincle–Fcγ–Syk and TLR2 signalling. (a) WT and TLR2^–^/^–^ BMDMs (10^7^) were infected with *M. bovis* BCG (MOI 10) or left untreated for 24 h. Cells were lysed and 4 µg cellular proteins were analysed by Western blot to assess Mincle expression. β-Actin expression was used as loading control. Western blot was performed once with combined lysates from two independent experiments. WT, Mincle^–^/^–^ and Fcγ^–^/^–^ (b), WT and IL-10R^–^/^–^ (c) or WT and TLR2^–^/^–^ BMDMs (10^5^) were infected with *M. bovis* BCG (MOI 10) or stimulated with LPS (100 ng/ml) for 48 h. Where indicated (b), BMDMs were incubated with 200 nM Syk inhibitor for 30 min prior to infection with *M. bovis* BCG. IL-10 or IL-12p40 concentrations were measured in BMDM supernatants by ELISA. Data are expressed as means ± SEM from three (b, c and d) independent experiments done in triplicate (one-way ANOVA followed by Bonferroni’s post-test, *P < 0.05; **P < 0.01; ***P < 0.001).

As resting macrophages express only minuscule amounts of Mincle,^5^ we analysed whether BCG can induce Mincle expression in a TLR2-dependent manner. BCG enhanced Mincle expression similarly to control cells exposed to LPS but not TLR2 ligands (Figure 2A).

### Mincle is involved in mycobacteria-induced IL-10 response without interfering with IL-12p40 production

IL-10 and IL-12p40 production were analysed in WT, Mincle^–/–^, Fcγ^–/–^ and Syk inhibitor-treated BMDMs exposed to BCG. In the absence of either Mincle, Fcγ or upon Syk inhibition, IL-10 response to BCG was significantly reduced, whereas IL-12p40 secretion was comparable (Figure 2B). However, significantly more IL-12p40 was produced upon BCG infection in IL-10R^–/–^ when compared with WT BMDMs (Figure 2C).

To investigate whether TLR2 is involved in IL-10 induction, WT and TLR2^–/–^ BMDMs were exposed to live BCG. IL-10 production was only partially reduced, whereas generation of IL-12p40 was signicantly enhanced in TLR2-deficient macrophages (Figure 2D).

In summary, Mincle is critical for the induction of IL-10 in macrophages by mycobacteria, whereas TLR2 is required for induction and inhibition of IL-10 and IL-12p40, respectively.

## Discussion

Here we report that Mincle signalling triggers macrophage IL-10 production, which subsequently modulates IL-12p40 secretion in an autocrine manner. Thus, besides its well-known pro-inflammatory properties, Mincle also modulates innate immunity to mycobacteria.

TDM and TLR2 ligands synergistically induced both, pro- and anti-inflammatory responses by macrophages, that is, TNF-α, NO and IL-10, in line with previous reports.^6,11^ In contrast, we found dampening of TLR2-mediated IL-12p40 secretion by TDM-coated beads similar to fungal dectin-1 ligands.^12^ Interestingly, induction of IL-10 by TDM was abolished upon interference with Mincle or Syk function. Our data are consistent with previous reports on the essential role of Syk in mediating TDM/Pam_3_CSK_4_-induced IL-10 in neutrophils but further show that Mincle is essential for induction of IL-10 in macrophages.^11^ In contrast, lack of Mincle or inhibition of Syk rescued IL-12p40 responses only to some extent, indicating that other anti-inflammatory factors may also contribute to the modulation of IL-12p40 production such as TGF-β. Up-regulation of Mincle was observed in mice infected with BCG.^7^ Accordingly, we revealed a significant up-regulation of Mincle in macrophages upon mycobacterial infection, independently of TLR2 but likely due to other PRRs such as MCL (CLEC4D).

Here, we show that Mincle is essential for macrophage IL-10 response to mycobacteria. Moreover, lack of Fcγ chain, as well as pharmacological inhibition of Syk, reduced IL-10 production by BCG-infected macrophages. These results extend previous studies showing a role of signalling molecules downstream of Mincle, that is, Fcγ chain or Syk, in pro-inflammatory cytokine induction by BCG.^4^

Despite IL-10-mediated impairment of IL-12p40 production by BCG, our data indicate that Mincle is not solely responsible for mycobacterial interference with pro-inflammatory cytokine secretion. Owing to the higher complexity of interactions between mycobacterial PAMPs with macrophages when compared with the simplistic TDM bead model, this is no surprise. TLR2-deficient macrophages produced no or less IL-10 than WT cells upon TDM-bead (Figure S2) or BCG infection, respectively, while still secreting significant amounts of IL-12p40. Notably, neither IL-10 nor IL-12p40 production was affected by the lack of NOD2 (Figure S3). Our data are in line with previous reports showing TLR2-mediated induction of IL-10 in macrophages and neutrophils by mycobacteria.^13,14^

Taken together, we demonstrate herein an important function of Mincle in modulating innate immune responses to mycobacteria through induction of the anti-inflammatory cytokine IL-10. These findings are of broader relevance for bacterial infections as indicated by a recent report on Mincle mediated IL-10 induction by *Helicobacter pylori*.^15^ Induction of IL-10 requires synergy between Mincle and TLR2 through mycobacterial ligands, TDM and lipoproteins, respectively. Moreover, Mincle is involved in IL-10-dependent down-regulation of TLR2-mediated IL-12p40 production. As blocking IL-10 enhances protection by BCG vaccination against *M. tuberculosis* infection,^16^ our findings indicate that interference with Mincle signalling can improve the protective capacity of BCG or TDB-containing adjuvant-based subunit vaccines against TB.
